# Risk factors associated with sickle cell retinopathy: findings from the Cooperative Study of Sickle Cell Disease

**DOI:** 10.1186/s40942-022-00419-8

**Published:** 2022-09-22

**Authors:** Mohammed Nawaiseh, Allaa Roto, Yara Nawaiseh, Mohammad Salameh, Rund Haddadin, Lana Mango, Hussam Nawaiseh, Doha Alsaraireh, Qais Nawaiseh, Saif Aldeen AlRyalat, Amer Alwreikat, David J. Ramsey, Nakhleh Abu-Yaghi

**Affiliations:** 1 King Hussein Cancer Center, Amman, Jordan; 2 Al Bahar Eye center, Ibn Sina Hospital, Ministry of Health, Kuwait city, Kuwait; 3 Faculty of Medicine, Jordan University of Science and Technology, Irbid, Jordan; 4 Hamad Medical Corporation, Doha, Qatar; 5 School of Medicine, The University of Jordan, Amman, Jordan; 6 Department of Special Surgery, Ophthalmology Division, School of Medicine, The University of Jordan, P.O. Box: 7599, Amman, 11118 Jordan; 7 Division of Ophthalmology, Department of Surgery, Lahey Hospital & Medical Center, Burlington, MA USA; 8 Department of Ophthalmology, Tufts University School of Medicine, Boston, MA USA

**Keywords:** Sickle cell disease, Sickle cell retinopathy, Proliferative sickle cell retinopathy, Hemoglobin F, Smoking

## Abstract

**Background:**

Sickle cell retinopathy (SCR) is one of the most important ocular manifestations of sickle cell disease (SCD). This study aims to assess the prevalence of SCR in SCD, identify risk factors for its development and progression to proliferative sickle cell retinopathy (PSCR), and evaluate the potential implications of these results on clinical practice.

**Methods:**

This research is a secondary analysis of patients diagnosed with SCD from the epidemiological, multicenter Cooperative Study of Sickle Cell Disease (CSSCD). We included all patients who completed a full ophthalmic evaluation. We identified clinical and laboratory SCD characteristics associated with SCR using multivariate logistic regression models. Proliferative sickle cell retinopathy (PSCR) was diagnosed according to the Goldberg classification system.

**Results:**

Of the 1904 study participants with SCD who met the inclusion criteria, 953 (50.1%) had retinopathy; of which 642 (67.3%) had bilateral disease. SCR was associated with older age (p < 0.001), history of smoking (p = 0.001), hematuria (p = 0.050), and a lower hemoglobin F (HbF) level (p < 0.001). PSCR risk increased with smoking (p = 0.005), older age (p < 0.001) higher hemoglobin level (p < 0.001) and higher white blood cell count (p = 0.011). Previous blood transfusion (p = 0.050), higher reticulocyte count (p = 0.019) and higher HbF level (p < 0.001) were protective factors against the development of PSCR. Ocular symptoms were associated with progression to PSCR in patients with SCR (p = 0.021).

**Conclusion:**

In this cohort of individuals with SCD, half of the participants had signs of SCR. Smoking and blood hemoglobin level were the two modifiable risk factors associated with increased retinopathy progression. Screening to identify the different stages of retinopathy, actively promoting smoking cessation, and optimizing the hematological profile of patients with SCD should guide treatment protocols designed to prevent the vision-threatening complications of the disease.

**Supplementary Information:**

The online version contains supplementary material available at 10.1186/s40942-022-00419-8.

## Introduction

Sickle cell disease (SCD) is an inherited group of hemoglobinopathies. Affected individuals suffer damage to organs and tissues from erythrocytes that structurally deform under hypoxic conditions because of variants in the beta-globin gene [1]. This leads to vaso-occlusions, endothelial cell activation, inflammation, and oxidative stress [2, 3]. Sickle cell retinopathy (SCR) is one of the most serious ocular manifestations of SCD. This condition is caused by the accumulated damage to the microcirculation of the retina leading to ischemic maculopathy and peripheral occlusions [2–4]. The clinical manifestations of SCR are broadly divided into non-proliferative sickle cell retinopathy and proliferative sickle cell retinopathy (PSCR), which is characterized by the development of retinal neovascularization and fibrovascular proliferation [5, 6].

SCD causes significant morbidity and mortality, affecting 70,000–100,000 people in the United States [7]. The incidence of SCD is approximately 0.4% among black individuals in North America, with approximately 8% of this population carrying sickle cell trait (AS) [8]. SCR in the U.S. occurs in 15–20% of patients homozygous for the hemoglobin S (SS) genotype and 33–40% of those who are heterozygous for the hemoglobin S and C genotypes, the so-called SC variant of the disease [9].

Ocular complications of SCD, including SCR and PSCR, have been shown to increase with age, generally being more common in adults than children [10–12], and, at least among those with the SS genotype, affect more males compared with females [13]. However, risk factors for development of SCR and its progression to PSCR remain poorly understood. The purpose of this study is to assess the prevalence of SCR in a large cohort of patients with SCD and identify hematologic and socio-medical factors associated with the development of SCR and its progression to proliferative disease. Understanding these risk factors may aid in the development of treatment and prevention strategies aimed at preventing the vision-threatening complications of the disease.

## Methods

### Study design

This study is a secondary analysis of data obtained from the epidemiological, multicenter Cooperative Study of Sickle Cell Disease (CSSCD), which was conducted from 1977 to 1995. Study enrollment ended in 1988, and it involved data collection at 23 university-based institutions in a uniform, standardized fashion. The study aimed to determine the natural history and clinical course of SCD from birth to death in order to identify those factors likely contributing to the morbidity and mortality of the disease. The methods for CSSCD have been described previously [14, 15]. Included in our analysis are all participants enrolled in the CSSCD who had completed an ophthalmic evaluation, which was conducted for participants five years of age and older. In the original investigation, ophthalmological evaluations were performed by trained ophthalmologists under standardized conditions only once upon entry, or at the second annual visit, or upon study exit, without any follow-up evaluations. Ocular assessment of symptoms included reports of double vision, eye pain, vision loss, or blurred vision in either eye. Slit lamp and fundus examination included inspection of the conjunctiva, iris, choroid, retina, macula, optic disc and vitreous.

The presence of SCR was defined as having any of the following findings on ophthalmic examination: “salmon patch” hemorrhages in the periphery of the retina, characteristic retinal pigmentary changes or iridescent spots, sunbursts, vessel tortuosity, retinoschisis cavities, angioid streaks, macular vascular occlusions, ischemic changes in the choroid, optic disc-comma signs, central retinal artery or vein occlusions or any of the signs of PSCR [14–16]. Patients were classified as having SCR if they had at least one retinal finding. PSCR was defined according to the Goldberg classification system if any of the following additional findings were noted on examination by a retinal specialist: peripheral arterial occlusions, arteriolar-venular anastomoses, fibrovascular proliferation, retinal detachment, macular hemorrhage, neovascularization at the optic disc, pre-retinal or vitreous hemorrhage [16].

At study entry, all participants underwent physical and laboratory-based examination. Prior medical history was collected, including any history of blood transfusions. Annual follow up was arranged to collect prospective biometric data from all participants, including reports of any SCD crises or other acute complications associated with the disease. Clinical evaluation at each follow-up visit also included a general physical examination, complete blood count (CBC), assessing white blood cell (WBC), red blood cell (RBC), reticulocyte, and platelet counts, as well as hemoglobin and hemoglobin F (HbF) levels. RBC, WBC, reticulocyte and platelet counts were not collected if a patient was transfused within six months prior to the study visit. When available, the mean value of these laboratory variables was calculated from the entry visit to the fourth annual visit. Pediatric subjects were those who were younger than 18 years of age at the time of their entry visit.

Factors examined for an association with the development of SCR and PSCR included: gender, hemoglobin genotype, blood pressure (BP), body mass index (BMI), the presence of systemic SCD complications (e.g., seizure, cerebrovascular accident [CVA], hematuria, nephrotic syndrome, hearing loss, heart disease, hand foot syndrome, spleen infarction, pneumonia, lung infarction, leg ulcers, or previous painful crisis), smoking history (including the use of cigarettes, cigars, and/or pipes) and hematological laboratory testing (e.g., WBC, platelets, and reticulocyte counts, hemoglobin, and HbF levels) [10, 17–22]. BP was considered to be abnormal if systolic pressure was ≥ 130 mmHg or diastolic pressure was ≥ 90 mmHg. BMI ≥ 25 kg/m^2^ was considered overweight/obese.

### Hemoglobin genotype classification

Hemoglobin genotype was classified broadly into two groups according to the similarity of clinical manifestations [10, 13, 18, 19]. The sickle cell anemia (SCA) group included SS, Sβ0 thalassemia and SS with α thalassemia genotypes. The variant genotypes group included SC, Sβ + thalassemia, and other less common hemoglobin genotypes. Multiple studies have indicated that PSCR risk factors differ between SCA and variant genotypes [10, 18, 19]. Thus, a separate sub-analysis was conducted to assess the risk of PSCR among SCA and variant genotypes.

### Data collection and ethical approval

Approval to access the CSSCD study data was obtained from the BioLINCC (Biologic Specimen and Data Repository Information Coordinating Center; https://biolincc.nhlbi.nih.gov/home/) [23], which is an open access data repository. This study received an official waiver of ethical approval from the institutional review board (IRB) at Jordan University Hospital, Amman, Jordan (waiver # 10/2022/7482).

### Statistical analysis

Categorical variables were compared using the two-sided chi-square test. Data for continuous variables were recorded as median (interquartile range [IQR]) and compared by using the Mann–Whitney U test. All tests were two-sided, and p-values below 0.05 were considered statistically significant. Predictors identified in the univariate analyses found to be associated with SCR or PSCR were entered into a multivariable logistic regression model and backward stepwise regression analyses technique to identify important covariates. Odds ratios (ORs) and 95% confidence intervals (CIs) were calculated for each variable. The final models included all variables significant at the 0.05 level (SPSS® Statistics for Windows, version 25.0, IBM Corp., Armonk, NY).

## Results

Of the 1904 participants with SCD who met the inclusion criteria, slightly more than half were female (55.2%, n = 1051). The vast majority of participants enrolled were black (97.9%). The median age at entry visit for the whole cohort was 17.0 years (IQR = 10.0–26.0). Pediatric patients comprised 55.3% of participants with a median age at entry of 11.0 years (IQR = 7.0–15.0). Adults comprised 44.7% of participants with a median age at entry of 27 years (IQR = 23–33). Ophthalmic evaluation was completed after a median of 232 days (IQR = 52–515) after entry into the study. Finally, 26.2% participants had a variant hemoglobin genotype (SC, Sβ + , other). Almost exactly half of subjects had SCR (50.1%) and bilateral disease was noted in 67.3% of those individuals. Pediatric and adult patients had similar frequency of bilateral SCR involvement (67.9% vs. 69.5%, p = 0.292). However, PSCR was more likely to be bilateral in adults (71.8%) compared to pediatric patients (48.2%, p < 0.001).

Table 1 summarizes the demographic and clinical characteristics of the study cohort and presents their association with SCR and PSCR. SCR was more common among adults compared with pediatric patients included in the study cohort (59.5% versus 40.5%, p < 0.001). Similarly, PSCR was more common in adults than in children (74.5% versus 25.5%, p < 0.001). Males were more likely to present with SCR (48.2%) compared with those without SCR (41.3%, p = 0.003). By contrast, females were less likely to have SCR (51.8%) compared with those without SCR (58.7%, p = 0.003). These same associations held true for PSCR. Variant genotypes were more likely to present with SCR and PSCR compared with SCA genotypes (71.7% versus 28.3% [p = 0.041] and 60.0% versus 24.2% [p < 0.001], respectively). Smoking, the presence of eye symptoms, BP ≥ 130/90 (mmHg), and BMI ≥ 25 (kg/m^2^) were all associated with higher prevalence of SCR and PSCR.

**Table 1:** Demographic and Clinical Characteristics of Study Cohort

Characteristic	Whole cohort	SCR			PSCR		
	n = 1904	Yes (n = 953) n (%)	No (n = 951) n (%)	*P*-value^a^	Yes (n = 423) n (%)	No (n = 1481) n (%)	*P*-value^a^
Age at entry							
Median (years)^b^	17.0 (10.0–26.0)	22.0 (14.0–29.0)	13.0 (7.0–21.0)	** < 0.001**	25.5 (18.0–32.0)	15.0 (8.0–23.0)	** < 0.001**
Adult	852 (44.7%)	567 (59.5%)	285 (30.0%)	** < 0.001**	315 (74.5%)	537 (36.3%)	** < 0.001**
Pediatric	1052 (55.3%)	386 (40.5%)	666 (70.0%)		108 (25.5%)	944 (63.7%)	
Sex							
Female	1051 (55.2%)	493 (51.8%)	558 (58.7%)	**0.003**	213 (50.5%)	838 (56.6%)	**0.025**
Male	851 (44.7%)	458 (48.2%)	393 (41.3%)		209 (49.5%)	642 (43.4%)	
Race							
Black	1864 (97.9%)	937 (98.8%)	927 (97.9%)	0 .103	419 (99.8%)	1445 (98.0%)	0.101
Other races	31 (1.6%)	11 (1.2%)	20 (2.1%)		1 (0.2%)	30 (2.0%)	
Smoking	588 (30.9%)	400 (44.9%)	188 (25.6%)	** < 0.001**	216 (53.1%)	372 (30.6%)	** < 0.001**
Eye symptoms	446 (23.4%)	258 (27.1%)	188 (19.8%)	** < 0.001**	146 (34.5%)	300 (20.3%)	** < 0.001**
BP ≥ 130/90 (mmHg)	152 (8.0%)	101 (10.8%)	51 (5.6%)	** < 0.001**	55 (13.2%)	97 (6.8%)	** < 0.001**
BMI ≥ 25 (kg/m^2^)	183 (9.6%)	116 (12.7%)	67 (7.4%)	** < 0.001**	67 (16.5%)	116 (8.2%)	** < 0.001**
Genotype							
SCA^c^	1403 (73.7%)	682 (71.7%)	719 (75.8%)	**0.041**	253 (60.0%)	1148 (77.7%)	** < 0.001**
Variant^d^	501 (26.3%)	269 (28.3%)	229 (24.2%)		169 (40.0%)	329 (22.3%)	

*SCR* sickle cell retinopathy, *PSCR* proliferative sickle cell retinopathy, *BMI* body mass index, *BP* blood pressure

Significant associations are marked in bold (p < 0.05)

^a^*P*-values determined using chi-square test for categorical variables and Mann–Whitney *U* test for continuous variables (age at entry)

^b^Median (interquartile range)

^c^Sickle cell anemia (SCA) genotypes include SS, Sβ0 and SSα

^d^Variant genotypes include SC, Sβ + , other

Table 2 evaluates the potential risk factors associated with the development of SCR and PSCR. Of the clinical variables collected during the course of the study, a history of aseptic necrosis, hematuria, spleen infarction, pneumonia, leg ulcers, painful crisis, and prior blood transfusions were all associated with a higher prevalence of SCR. Among the laboratory data collected, higher hemoglobin levels were associated with SCR, while lower hemoglobin F levels, platelet, and reticulocyte counts were all less likely to be associated with SCR. Development of PSCR shared the same associations apart from splenic infarction. In addition, hand foot syndrome and higher WBC count were found to be associated with the development of PSCR.

**Table 2:** Clinical and laboratory variables associated with SCR and PSCR

Characteristic	Whole cohort	SCR			PSCR		
	n = 1904	Yes (n = 953) n (%)	No (n = 951) n (%)	*P*-value^a^	Yes (n = 423) n (%)	No (n = 1481) n (%)	*P*-value^a^
Previous history of							
Seizure	150 (7.9%)	68 (7.2%)	82 (8.6%)	0.234	30 (7.1%)	120 (8.1%)	0.502
CVA	134 (7.0%)	59 (6.2%)	75 (7.9%)	0.152	24 (5.7%)	110 (7.4%)	0.217
Aseptic necrosis	334 (17.5	208 (21.9%)	126 (13.2%)	** < 0.001**	114 (27.0%)	220 (14.9%)	** < 0.001**
Hematuria	250 (13.1%)	155 (16.7%)	95 (10.5%)	** < 0.001**	76 (18.4%)	174 (12.2%)	**0.001**
Nephrotic syndrome	68 (3.6%)	37 (4.0%)	31 (3.4%)	0.528	15 (3.6%)	53 (3.7%)	0.921
Hearing loss	116 (6.1%)	62 (6.6%)	54 (5.9%)	0.534	29 (7.0%)	87 (6.1%)	0.516
Heart disease	246 (12.9%)	133 (14.3%)	113 (12.4%)	0.239	62 (14.9%)	184 (12.9%)	0.292
Hand foot syndrome	617 (32.4%)	296 (35.3%)	321 (37.5%)	0.342	110 (29.9%)	507 (38.2%)	** < 0.001**
Spleen infarction	163 (8.6%)	68 (7.5%)	95 (10.8%)	**0.016**	38 (9.4%)	125 (9.1%)	0.836
Pneumonia	1045 (54.9%)	551 (59.7%)	494 (54.4%)	**0.022**	254 (61.5%)	791 (55.8%)	**0.039**
Lung infarction	86 (4.5%)	50 (5.5%)	36 (4.0%)	0.148	26 (6.4%)	60 (4.3%)	0.076
Leg ulcers	235 (12.3%)	147 (15.7%)	88 (9.6%)	** < 0.001**	77 (18.5%)	158 (11.0%)	** < 0.001**
Painful crisis	975 (51.2%)	518 (55.0%)	457 (48.4%)	**0.004**	237 (56.8%)	738 (50.2%)	**0.017**
Blood transfusion	1140 (59.9%)	617 (66.0%)	523 (57.3%)	** < 0.001**	274 (66.0%)	866 (60.4%)	**0.039**
Laboratory data							
Hemoglobin (g/dl)^b^	9.0 (8.0–10.6)	9.1 (8.0–10.8)	8.9 (8.0–10.2)	**0.008**	10.0 (8.4–11.5)	8.8 (7.9–10.1)	** < 0.001**
WBC (10^9^/L)^b^	10.8 (8.7–13.0)	10.7 (8.7–12.9)	10.9 (8.7–13.1	0.296	10.5 (8.1–12.7)	10.9 (8.9–13.1)	**0.002**
Platelets (10^9^/L)^b^	392.0 (300.0–480.2)	382.0 (296.0–465.3)	402.6 (302.9–494.7)	**0.008**	354.0 (266.7–439.5)	405.5 (310.8–487.5)	** < 0.001**
Reticulocytes (%)^b^	9.7 (5.5–13.8)	9.4 (5.2–13.4)	10.0 (5.9–14.1)	**0.020**	7.5 (4.3–11.6)	10.2 (6.2–14.2)	** < 0.001**
HbF (%)^b^	3.8 (2.0–7.6)	3.3 (1.7–6.0)	4.7 (2.3–9.0)	** < 0.001**	2.6 (1.2–4.5)	4.4 (2.2–8.5)	** < 0.001**

*SCR* sickle cell retinopathy, *PSCR* proliferative sickle cell retinopathy, *CVA* cerebrovascular accident, *WBC* white blood cells, *HbF* hemoglobin F

Significant associations are marked in bold (p < 0.05)

^a^*P*-values determined using chi-square test for categorical variables and Mann–Whitney U test for continuous variables (age at entry)

^b^Median (interquartile range)

Logistic regression analysis was used to identify variables that when combined predicted the presence of retinopathy in our study cohort. Variables from Tables 1 and 2 that were statistically significant (p < 0.05) in the univariate analysis for the development of SCR or PSCR were included in the multivariate logistic regression models, in a backward stepwise manner. The other demographic and clinical variables included in Tables 1 and 2 were excluded from our logistic regression model because they lacked unique predictive value regarding likelihood of retinopathy. The logistic regression model for SCR patients correctly classified 64.3% of cases (model *χ*^2^ [4] = 105.8, p < 0.001, Nagelkerke *R*^2^ = 0.13). The logistic regression model for PSCR patients correctly classified 78.6% of cases (model *χ*^2^ [7] = 191.4, p < 0.001, Nagelkerke *R*^2^ = 0.27).

PSCR risk increased with smoking (Odds ratio [OR] = 1.66, P = 0.005), older age (OR = 1.05, P < 0.001), higher hemoglobin level (OR = 1.34, P < 0.001) and higher WBC count (OR = 1.08, P = 0.011). Previous blood transfusion (OR = 0.64, P = 0.050), higher reticulocyte count (OR = 0.95, P = 0.019) and higher HbF level (OR = 0.86, P < 0.001) were protective factors against the development of PSCR. Older age at entry visit (OR = 1.04, P < 0.001), smoking (OR = 1.67, P = 0.001) and those with hematuria (OR = 1.53, P = 0.050) were more likely to develop SCR than patients with lower age, non-smokers and those without hematuria. On the other hand, higher HbF level (OR = 0.93, p < 0.001) was a protective factor against the development of SCR.

Additional File 1: Table S1 shows the univariate analysis of the characteristics of SCA and variant genotypes patients by PSCR status. Variables that were statistically significant (p < 0.05) in the univariate analysis were included in the multivariate binary logistic regression for the risk factors of PSCR among SCA and variant genotypes patients. The logistic regression model for PSCR among SCA genotypes correctly classified 79.6% of cases and was statistically significant (*χ*^2^ [5] = 115.1, p < 0.001, Nagelkerke *R*^2^ = 0.24). The logistic regression model for PSCR among variant genotypes correctly classified 71.4% of cases and was statistically significant (*χ*^2^ [5] = 59.8, p < 0.001, Nagelkerke *R*^2^ = 0.26).

Among patients with SCA genotypes, PSCR was more likely in older age (OR = 1.06, P < 0.001), smokers (OR = 1.73, P = 0.014), and higher hemoglobin level (OR = 1.27, P = 0.006). Higher reticulocyte count (OR = 0.93, P = 0.005) and higher Hb F level (OR = 0.86, P < 0.001) were protective factors against the development of PSCR in SCA genotypes. Among patients with variant genotypes, PSCR was more likely in older age (OR = 1.04, P < 0.001), those with eye symptoms (OR = 1.78, P = 0.050), those with higher hemoglobin level (OR = 1.54, P < 0.001), and those with higher WBC count (OR = 1.11, P = 0.049). Higher Hb F level (OR = 0.83, P = 0.005) was protective factors against the development of PSCR (Table 3).

**Table 3:** Multivariate logistic regression analysis of variables associated with PSCR and SCR for patients in the study cohort

	OR	95% CI	P-value
SCR	n = 953		
Age at entry (years)	1.04	1.02, 1.05	< 0.001
Smoking	1.67	1.22, 2.28	0.001
Previous history of hematuria	1.53	1.01, 2.23	0.050
HbF (%)	0.93	0.91, 0.96	< 0.001
PSCR	n = 423		
Age at entry (years)	1.05	1.03, 1.07	< 0.001
Smoking	1.66	1.16, 2.37	0.005
Previous history of blood transfusion	0.64	0.41, 0.99	0.050
Hemoglobin (g/dl)	1.34	1.17, 1.54	< 0.001
WBC (10^9^/L)	1.08	1.02, 1.15	0.011
Reticulocytes (%)	0.95	0.91, 0.99	0.019
HbF (%)	0.86	0.81, 0.91	< 0.001
PSCR among SCA^a^ genotypes	n = 253		
Age at entry (years)	1.06	1.03, 1.08	< 0.001
Smoking	1.73	1.11, 2.67	0.014
Hemoglobin (g/dl)	1.27	1.07, 1.50	0.006
Reticulocytes (%)	0.93	0.89, 0.98	0.005
HbF (%)	0.86	0.81, 0.92	< 0.001
PSCR among variant^b^ genotypes	n = 169		
Age at entry (years)	1.04	1.01, 1.07	< 0.001
Eye symptoms	1.78	1.02, 3.21	0.050
Hemoglobin (g/dl)	1.54	1.24, 1.91	< 0.001
WBC (10^9^/L)	1.11	1.01, 1.22	0.049
HbF (%)	0.83	0.72, 0.94	0.005

*SCR* sickle cell retinopathy, *PSCR* proliferative sickle cell retinopathy, *SCA* sickle cell anemia, *WBC* white blood cells, *HbF* hemoglobin F, *OR* odds ratio, *CI* confidence interval

^a^Sickle cell anemia (SCA) genotypes include SS, Sβ0 and SSα

^b^Variant genotypes include SC, Sβ + , other

Factors that were found to be associated with PSCR among the cohort of patients with SCR in the univariate analysis were higher age, smoking, eye symptoms, BP ≥ 130/90 (mmHg), BMI ≥ 25 (kg/m^2^), variant genotype, aseptic necrosis, hand foot syndrome, spleen infarction, leg ulcers, higher hemoglobin, lower WBC, platelet, and reticulocyte counts, and lower HbF levels (Additional File 2: Table S2). These variables were entered into a multivariate logistic regression model. Patients with SCR identified to have PSCR were more likely to be older (OR = 1.06, P < 0.001), report eye symptoms (OR = 1.68, P = 0.021), have higher hemoglobin levels (OR = 1.29, P < 0.002), higher WBC counts (OR = 1.11, P = 0.004), lower reticulocyte counts (OR = 0.96, P = 0.050), and lower HbF levels (OR = 0.90, P < 0.001, Table 4). A logistic regression model assessing all of the factors associated with the development of PSCR correctly classified 68.3% of cases (model *χ*^2^ [6] = 122.3, P < 0.001, Nagelkerke *R*^2^ = 0.26).

**Table 4:** Multivariate logistic regression analysis of variables associated with the development of PSCR among patients with SCR

PSR	n = 423		
	OR	95% CI	P-value
Age at entry (years)	1.06	1.04, 1.08	< 0.001
Eye symptoms	1.68	1.08, 2.62	0.021
Hemoglobin (g/dl)	1.29	1.14, 1.47	< 0.001
WBC (10^9^/L)	1.11	1.03, 1.19	0.004
Reticulocytes (%)	0.96	0.91, 0.99	0.050
HbF (%)	0.90	0.85, 0.94	< 0.001

*SCR* sickle cell retinopathy, *PSCR* proliferative sickle cell retinopathy, *OR* odds ratio, *CI* Confidence interval, *WBC* white blood cells, *HbF* hemoglobin F

## Discussion

The epidemiologic CSSCD study provides one of the largest data sets to assess risk factors associated with development of SCR and PSCR. SCR is a common problem among patients with SCD affecting more than half of this population. Not surprisingly, adults were more likely to be identified to have SCR compared with pediatric patients. SCR was also slightly more common in males than in females. Notably, two thirds of patients identified to have SCR had bilateral disease, and this finding was also more common in adults than among pediatric patients. Finally, SCR was found to be associated with older age, history of smoking, hematuria, and a lower HbF level. Tobacco smoking is an important, modifiable risk factor for morbidity in SCD and for the development of SCR [24, 25]. Smoking cessation counseling and tobacco dependence interventions should therefore be considered to reduce the burden of the eye disease.

PSCR risk increased with smoking and higher age, hemoglobin level and WBC count. Previous blood transfusion, higher reticulocyte count, and higher HbF level were protective factors against the development of PSCR. Furthermore, eye symptoms were associated with progression to PSCR in patients with SCR. Eye symptoms, such as eye pain and blurred vision, should be an indication for retinal re-examination, especially in variant patients who are more prone to vaso-occulsive events [26]. This provides an important reminder to patients and care providers that all patients need routine eye examinations, but those who have risk factors or symptoms may require more frequent eye checks [9, 19, 27].

We found that the risk of SCR, and especially PSCR, increases with advancing age. SCR is a disease that often begins in childhood with PSCR more commonly identified by early adulthood [10–12], as was the case in our study cohort. PSCR likely follows from the accumulation of retinal damage from repeated occult vaso-occlusive episodes, which induce vascular endothelial growth factor secretion from ischemic retinal tissue [9, 13, 18, 19]. Downes et al*.* in a prospective longitudinal 20-year observational study, showed an increasing incidence of PSCR with age, with more frequent development unilaterally than bilaterally [12]. By contrast, Fox et al*.* found that proliferative disease was more often bilateral, which mirrors the findings in our cohort [18].

The results in our univariate analysis indicate that SCR was more common in males than in females, which is in line with several previous studies [9, 13, 19, 27]. After accounting for other factors, we did not identify male sex as an independent risk factor for the development of SCR or PSCR. Potential reasons for this may stem from the fact that smoking, which promotes the development of SCR, is more prevalent among men than women [28]. Smoking is associated with chronic vasculopathy, increases the risk of pneumonia, and induces states of general acidosis and hypoxia in the body. This in turn causes more sickling episodes and vaso-occlusive events leading to an increased risk of going on to develop more severe stages of SCR and proliferative disease [24, 25]. In our study population males also had a lower level of HbF level compared with females (median of 3.3% [IQR = 1.6–6.4%] versus 4.4% [IQR = 2.1–8.3%, p < 0.001), which is consistent with other studies that reported higher HbF levels in females among patients with SCD [29–31]. Finally, although women may be relatively protected from progression to SCR because of the well-known effects of estrogen stimulating nitric oxide synthesis and mediating anti-inflammatory and anti-apoptotic signaling pathways, which could protect vessels and tissues during SCD crises [32], our study included many younger individuals who would not yet have benefited from the higher estrogen levels after puberty.

Our study examined vaso-occlusive episodes and their consequences, e.g. aseptic necrosis, leg ulcers, hematuria, and painful crisis. A majority of these factors were significantly associated with SCR and PSCR in univariate analyses. However, in our model only hematuria remained associated with an increased risk of developing SCR. In the kidneys, the hypoxic environment of the renal medulla predisposes for RBCs sickling and vaso-occlusion, which causes renal papillary necrosis and hematuria [33]. In the retina, vaso-occlusive episodes cause hypoxia, ischemia, and local tissue damage when sickled RBCs occlude small blood vessels. This promotes inflammation, thrombosis and reactive blood vessel formation in the form of neovascularization, thereby increasing the risk of PSCR [34, 35]. Further research is warranted to investigate the connection between the kidney and the retina in SCD. Although, a previous report from the CSSCD had found an association between eye diseases and neurological manifestation of SCD, such as seizures and strokes, we did not find an association between the neurological manifestations of SCD and the development of SCR [36].

By looking at hematological indices, we found that higher hemoglobin levels increased the risk of PSCR. This was true for individual with both the SCA and variant genotypes. One possibility is that this effect may be confounded by chronic transfusion, which we are unable to account for as there was no data on the frequency or timing of any blood transfusions. Excessive chronic transfusions are associated with hyperviscosity, micro-hemolysis, and iron toxicity, all of which can worsen retinopathy. Thus, more research is warranted to determine an optimal hemoglobin level and to optimize transfusion strategies. Similar to our results, Fox et al*.* found that SCR risk in SS genotype was increased with a higher total hemoglobin level in males [18]. On the contrary, other studies, which better accounted for chronic transfusions, found no association between hemoglobin level and retinopathy [19, 27].

We found that elevated WBC counts were associated with increased risk of PSCR. The relationship between higher WBC counts and retinopathy is not yet well understood. It was found that a higher number of intraretinal polymorphonuclear leucocytes increased the disease progression and that leucocyte adhesion molecule might play an important part in the vaso-occlusive phase of SCR by adhering to blood vessel walls and aggregating with other blood cells with more effective blockage of the lumen [37, 38]. Leukocyte count reduction and targeted blockade of specific leukocyte adhesion molecules might play a role in protecting against SCR. Contrary to our results, Estepp et al*.* found that there was no association between WBC counts and retinopathy in children [39].

Higher HbF level was protective against the development of SCR and PSCR. A low HbF level has previously been reported to be associated with PSCR [18, 27, 40]. HbF acts to dilute the amount of HbS available for sickling, thus decreasing the severity of tissue ischemia and inflammation under hypoxic conditions. Hydroxyurea, which stimulates HbF production in SCD, may play a protective role against SCR [39, 41, 42]. More research is warranted to explore this protective effect of hydroxyurea on the development of SCR. We found that higher reticulocyte counts were protective against PSCR. Increased reticulocyte counts demonstrate a healthy bone marrow response to hemolytic episodes. Reticulocytes are produced in response to erythropoietin secreted from the kidney in conditions of hypoxia [43]. These two hematologic factors, higher reticulocytes and HbF, work together to protect the whole body from severe ischemia and hypoxia, consequently decreasing the tendency for PSCR to develop.

Previous studies have found that patients with variant genotypes appear to be at increased risk of SCR and PSCR compared with those with the SCA genotypes [10, 13, 18–20, 44]. Our study found a similar association in a univariate analysis, but, after accounting for other factors, genotype was not significantly associated with retinopathy or more severe eye disease. Nevertheless, individuals with the SS genotype may be more prone to new vessel formation and tend to have more auto-infarction events [11]. This effect could cause regression of abnormal vessels or inhibit the further growth or branching of new vessels, thus stifling the development of proliferative changes. By contrast, the SC variant appears to cause fewer and less extensive vaso-occlusive episodes, which may allow neovascularization to continue uninterrupted [19, 27]. Another mechanism proposed for why more severe disease is associated with the SC variant compared with SS disease relates to the fact that hemoglobin C is associated with an increase in the activity of K:Cl co-transporter. Loss of K^+^ through this cotransporter promotes RBC dehydration, which contributes to sickling of the HbS molecules. This likely accounts for the increased frequency of vaso-occlusion events in patients with the SC variant and thus higher likelihood of PSCR development [26].

Our research identifies a potential benefit from prior blood transfusions on the risk of developing PSCR. This could help patients by diluting the concentration of sickled RBCs in the blood. However, blood transfusions are used less frequently today because of the risks associated with iron overload. We are also careful not to draw too strong a conclusion from this finding because our available study data did not provide information concerning the frequency, number, or timing of transfusion events, especially in relation to the timing of the ocular examination. Interestingly, Hasan et al*.* noted that the frequency of retinopathy was higher in patients who started transfusions at an older age [45].

The findings of this research must be viewed in the light of some limitations, including the historic nature of the large CSSCD study. Although eye examinations were standardized and collected on study entry, only one ocular examination was recorded for each patient without any follow up of disease progression. The accuracy of these examinations was enhanced by the prospective collection of this data by ophthalmology specialists and generalizability enhanced by sampling patients from different centers across the United States. However, limited data on other medical comorbidities and associated laboratory data were collected in the study. For example, important comorbidities such as diabetes mellitus and presence of diabetic retinopathy on retinal examination were not recorded. We are also unable to judge the effects of concurrent medical treatment with medications, like hydroxyurea or chelation therapy, on the development of SCR in our study. As mentioned, we also lack details on the frequency and timing of blood transfusions. Clinical trials should determine an optimal goal of hemoglobin level and optimize transfusion strategies in SCD. Future studies should also include hemoglobin A1c to assess the effect of diabetic retinopathy on patients with SCR [46]. Another limitation of the study is the lack of documentation of any retina alterations with fundus imaging. The lack of imaging data reduces the reliability and reproducibility of the retinal examinations performed in the CSSCD study. Furthermore, the definition of SCR used at the time of the study, which included angioid streaks, larger vessels occlusion, choroid ischemic changes, optic disc-comma signs, and vessel tortuosity are non-specific signs for SCR and could be caused by other conditions that were not accounted for in this study. In future, the application of wide-angle fundus imaging and fluorescein angiography could be considered to increase the accuracy of retinal assessment.

## Conclusion

Retinopathy is common in patients with SCD, with more than half of all eyes affected by the condition. SCR is more common among adult patients but also develops in a great many patients while they are still in the pediatric age range. Screening for different stages of retinopathy with regular eye examinations and efforts to increase awareness of concurrent risk factors for retinopathy progression among patients and those who care for them can guide preventive strategies and treatment protocols for the disease. Several risk factors capable of being modified are associated with development of SCR and its risk of progression to proliferative disease. In particular, promoting smoking cessation and efforts to optimize hematologic profiles are important for reducing the morbidity associated with the development of SCR. Knowledge of these findings should assist in the development of vision-saving preventative measures and models capable of predicting which patients with SCD are at greatest risk for the development of retinopathy.

## Supplementary Information

**Additional file 1: Table S1.** Characteristics of SCA and variant genotypes patients by PSCR Status.

**Additional file 2: Table S2. **Clinical and laboratory variables associated with development of PSCR among patients with SCR.

## Data Availability

The datasets used and/or analyzed in this study can be accessed from the BIOLINCC (Biologic Specimen and Data Repository Information Coordinating Center) website: (https://biolincc.nhlbi.nih.gov/studies/csscd/).

## Abbreviations

SCR: Sickle cell retinopathy
SCD: Sickle cell disease
PSCR: Proliferative sickle cell retinopathy
CSSCD: Cooperative Study of Sickle Cell Disease
CBC: Complete blood count
WBCs: White blood cells
RBCs: Red blood cells
HbF: Hemoglobin F
BP: Blood pressure
BMI: Body Mass Index
CVA: Cerebrovascular accident
SCA: Sickle cell anemia
BioLINCC: Biologic Specimen and Data Repository Information Coordinating Center
IQR: Interquartile range
OR: Odds ratio
Sβ^+^: β^+^-Thalassemia.
SSα: Hemoglobin α-thalassemia [Hemoglobin H disease]
Sβ^0^: β^0^-Thalassemia
HbS: Hemoglobin S
SS: Homozygous for the hemoglobin S genotype
SC: Heterozygous for the hemoglobin S and C genotypes
IRB: Institutional review board
