# Supplementary material for: Risk factors associated with sickle cell retinopathy: findings from the Cooperative Study of Sickle Cell Disease
Source: Int J Retina Vitreous. 2022 Sep 22;8:68. doi: 10.1186/s40942-022-00419-8 (PMC9502612; doi:10.1186/s40942-022-00419-8)
Supplement: Supplementary file 1 — Additional file 1: Table S1. Characteristics of SCA and variant genotypes patients by PSCR Status. [file 40942_2022_419_MOESM1_ESM.docx]

**Table S1: Characteristics of SCA and variant genotypes patients by PSCR Status**

| **CHARACTERISTIC** | ***SCA***^§^ | | | ***Variant***^‡^ | | |
| --- | --- | --- | --- | --- | --- | --- |
|  | ***PSCR*** | | | ***PSCR*** | | |
|  | **Yes**  **n (%)** | **No**  **n (%)** | ***P*-value^†^** | **Yes**  **n (%)** | **No**  **n (%)** | ***P*-value^†^** |
| Gender (female) | 124 (49.0%) | 653 (56.8%) | **0.024** | 89 (52.7%) | 185 (56.1%) | 0.470 |
| Gender (male) | 129 (51.0%) | 497 (43.2%) | **0.024** | 80 (47.3%) | 145 (43.9%) | 0.470 |
| Age at entry (years) | 25.0 (19.0-31.0) | 15.0 (8.0-23.0) | **<0.001** | 26.0 (17.0-33.5) | 13.0 (8.0-24.0) | **<0.001** |
| BMI ≥ 25 (kg/m²) | 28 (11.5%) | 73 (6.6%) | **0.009** | 39 (23.9%) | 43 (13.7%) | **0.005** |
| Smoking | 137 (55.7%) | 297 (31.3%) | **<0.001** | 79 (49.1%) | 75 (28.1%) | **<0.001** |
| Eye symptoms | 75 (29.6%) | 231 (20.1%) | **0.001** | 71 (41.8%) | 69 (20.8%) | **<0.001** |
| BP ≥130/90 (mmHg) | 31 (12.4%) | 66 (6.0%) | **<0.001** | 24 (14.4%) | 31 (9.7%) | 0.124 |
| Previous history of: |  |  |  |  |  |  |
| Seizure | 21 (8.3%) | 103 (9.0%) | 0.739 | 9 (5.3%) | 17 (5.2%) | 0.934 |
| CVA | 15 (5.9%) | 92 (8.0%) | 0.261 | 9 (5.3%) | 18 (5.5%) | 0.952 |
| Aseptic necrosis | 80 (31.6%) | 183 (15.9%) | **<0.001** | 34 (20.1%) | 37 (11.2%) | **0.007** |
| Hematuria | 41 (16.5%) | 131 (11.8%) | **0.042** | 35 (21.2%) | 43 (13.7%) | **0.036** |
| Nephrotic syndrome | 8 (3.2%) | 40 (3.6%) | 0.755 | 7 (4.3%) | 13 (4.2%) | 0.958 |
| Hearing loss | 20 (8.0%) | 74 (6.7%) | 0.458 | 9 (5.5%) | 13 (4.1%) | 0.504 |
| Heart disease | 49 (19.7%) | 160 (14.4%) | **0.039** | 13 (7.8%) | 24 (7.6%) | 0.941 |
| Hand foot syndrome | 80 (36.5%) | 449 (43.6%) | **0.050** | 30 (20.1%) | 58 (19.5%) | 0.880 |
| Spleen infarction | 17 (7.1%) | 98 (9.2%) | 0.306 | 21 (12.9%) | 27 (8.9%) | 0.175 |
| Pneumonia | 179 (71.9%) | 659 (59.7%) | **<0.001** | 75 (45.7%) | 132 (41.9%) | 0.422 |
| Lung infarction | 19 (7.8%) | 50 (4.6%) | **0.042** | 7 (4.3%) | 10 (3.2%) | 0.535 |
| Leg ulcers | 68 (27.0%) | 139 (12.4%) | **<0.001** | 9 (5.5%) | 19 (6.0%) | 0.804 |
| Painful crisis | 147 (58.6%) | 588 (51.4%) | **0.038** | 90 (54.2%) | 150 (46.3%) | 0.097 |
| Blood transfusion | 202 (80.8%) | 747 (66.8%) | **<0.001** | 72 (43.6%) | 119 (37.9%) | 0.223 |
| Laboratory: |  |  |  |  |  |  |
| Hemoglobin (g/dl)^¶^ | 8.8 (7.9-9.8) | 8.4 (7.7-9.2) | **<0.001** | 11.8 (10.9-12.9) | 11.2 (10.5-12.0) | **<0.001** |
| WBC (10^9^/L)^¶^ | 11.5 (10.0-13.5) | 11.7 (10.0-13.5) | 0.864 | 8.0 (6.4-9.9) | 7.3 (5.9-9.7) | **0.045** |
| Platelets (10^9^/L)^¶^ | 403.3  (334.3-490.3) | 429.3  (359.5-509.9) | **0.002** | 274.5  (190.7-351.8) | 269.6  (215.1-350.1) | 0.266 |
| Reticulocytes (%)^¶^ | 10.6 (7.8-13.8) | 11.7(8.6-15.4) | **<0.001** | 3.9 (3.0-5.3) | 3.7 (2.7-5.5) | 0.481 |
| HbF (%)^¶^ | 3.5 (2.0-5.9) | 5.2 (2.9-9.2) | **<0.001** | 1.4 (0.7-2.5) | 2.1 (1.1-3.8) | **<0.001** |

SCA: sickle cell anemia, PSCR: proliferative sickle cell retinopathy, BMI: body mass index, BP: blood pressure, CVA: cerebrovascular accident, WBC: white blood cells, HbF: hemoglobin F.
**^†^***P*-values determined using chi-square test for categorical variables and Mann–Whitney U test for continuous variables (age at entry, hemoglobin, WBC, platelets, reticulocytes, HbF). Signiﬁcant associations are marked in bold (p < 0.05).

^¶^Median (interquartile range)
^§^Sickle cell anemia (SCA) genotypes include SS, Sβ0 and SSα.
^‡^Variant genotypes include SC, Sβ+, other.
