# Supplementary material for: Risk factors associated with sickle cell retinopathy: findings from the Cooperative Study of Sickle Cell Disease
Source: Int J Retina Vitreous. 2022 Sep 22;8:68. doi: 10.1186/s40942-022-00419-8 (PMC9502612; doi:10.1186/s40942-022-00419-8)
Supplement: Supplementary file 2 — Additional file 2: Table S2. Clinical and laboratory variables associated with development of PSCR among patients with SCR. [file 40942_2022_419_MOESM2_ESM.docx]

**Table S2:** Clinical and laboratory variables associated with development of PSCR among patients with SCR.

| **Characteristic** | **PSCR** | | |
| --- | --- | --- | --- |
|  | **PSCR (n = 423)**  **n (%)** | **NPSCR (n = 530)**  **n (%)** | ***P*-value^†^** |
| Age at entry |  |  |  |
| Median (years)^¶^ | 25.5 (18.0- 32.0) | 18.0 (11.0-26.0) | **< 0.001** |
| Adult | 315 (74.5%) | 252 (47.5%) | **< 0.001** |
| Pediatric | 108 (25.5%) | 278 (52.5%) |  |
| Sex |  |  |  |
| Female | 213 (50.5%) | 280 (52.9%) | 0.451 |
| Male | 209 (49.5%) | 249 (47.0%) |  |
| Smoking | 216 (51.1%) | 184 (34.7%) | **< 0.001** |
| Eye symptoms | 146 (34.5%) | 112 (21.1%) | **< 0.001** |
| BP ≥130/90 (mmHg) | 55 (13.2%) | 46 (8.6%) | **0.037** |
| BMI ≥ 25 (kg/m²) | 67 (15.8%) | 49 (9.2%) | **0.002** |
| Genotype |  |  |  |
| SCA^§^ | 253 (59.8%) | 429 (80.9%) | **< 0.001** |
| Variant^‡^ | 170 (40.2%) | 101 (19.1%) |  |
| Previous history of: |  |  |  |
| Seizure | 30 (7.1%) | 38 (7.2%) | 0.965 |
| CVA | 24 (5.7%) | 35 (6.6%) | 0.555 |
| Aseptic necrosis | 114 (27.0%) | 94 (17.8%) | **< 0.001** |
| Hematuria | 76 (17.9%) | 79 (14.9%) | 0204 |
| Nephrotic syndrome | 15 (3.6%) | 22 (4.1%) | 0.619 |
| Hearing loss | 29 (6.8%) | 33 (6.2%) | 0.714 |
| Heart disease | 62 (14.6%) | 71 (13.3%) | 0.626 |
| Hand foot syndrome | 110 (26.0%) | 186 (35.0%) | **0.004** |
| Spleen infarction | 38 (8.9%) | 30 (5.6%) | **0.050** |
| Pneumonia | 254 (60.0%) | 297 (56.0%) | 0.315 |
| Lung infarction | 26 (6.1%) | 24 (4.5%) | 0.268 |
| Leg ulcers | 77 (18.2%) | 70 (13.2%) | **0.035** |
| Painful crisis | 237 (56.0%) | 281 (53.0%) | 0.326 |
| Blood transfusion | 141 (33.3%) | 177 (33.3%) | 0.984 |
| Laboratory data: |  |  |  |
| Hemoglobin (g/dl)^¶^ | 10.0 (8.4 -11.5) | 8.6 (7.7-10.1) | **< 0.001** |
| WBC (10^9^/L)^¶^ | 10.5 (8.1 -12.7) | 10.9 (9.0- 13.1) | **0.002** |
| Platelets (10^9^/L)^¶^ | 354.0 (266.7- 439.5) | 408.3 (326.7- 481.2) | **< 0.001** |
| Reticulocytes (%)^¶^ | 7.58 (4.3 -11.6) | 10.5 (6.7 -14.5) | **< 0.001** |
| HbF (%)^¶^ | 2.6 (1.2-4.5) | 3.8 (2.1-7.5) | **< 0.001** |

SCR: sickle cell retinopathy, PSCR: proliferative sickle cell retinopathy, NPSCR: non-proliferative sickle cell retinopathy, CVA: cerebrovascular accident, BMI: body mass index, BP: blood pressure, WBC: white blood cells, HbF: hemoglobin F.
**^†^***P*-values determined using chi-square test for categorical variables and Mann–Whitney U test for continuous variables (age at entry, hemoglobin, WBC, platelets, reticulocytes, HbF). Signiﬁcant associations are marked in bold (p < 0.05).

^¶^Median (interquartile range)
^§^Sickle cell anemia (SCA) genotypes include SS, Sβ0 and SSα.
^‡^Variant genotypes include SC, Sβ+, other.
